# Using DNA From Mothers and Children to Study Parental Investment in Children’s Educational Attainment

**DOI:** 10.1111/cdev.13329

**Published:** 2019-10-27

**Authors:** Jasmin Wertz, Terrie E. Moffitt, Jessica Agnew‐Blais, Louise Arseneault, Daniel W. Belsky, David L. Corcoran, Renate Houts, Timothy Matthews, Joseph A. Prinz, Leah S. Richmond‐Rakerd, Karen Sugden, Benjamin Williams, Avshalom Caspi

**Affiliations:** ^1^ Duke University; ^2^ King’s College London; ^3^ Columbia University

## Abstract

This study tested implications of new genetic discoveries for understanding the association between parental investment and children’s educational attainment. A novel design matched genetic data from 860 British mothers and their children with home‐visit measures of parenting: the E‐Risk Study. Three findings emerged. First, both mothers’ and children’s education‐associated genetics, summarized in a genome‐wide polygenic score, were associated with parenting—a gene–environment correlation. Second, accounting for genetic influences slightly reduced associations between parenting and children’s attainment—indicating some genetic confounding. Third, mothers’ genetics were associated with children’s attainment over and above children's own genetics, via cognitively stimulating parenting—an environmentally mediated effect. Findings imply that, when interpreting parents’ effects on children, environmentalists must consider genetic transmission, but geneticists must also consider environmental transmission.

Parents devote a great deal of time and effort to ensuring their children’s educational success. They read to their children, buy educational toys, monitor their children’s schoolwork, and enroll them in enriching classes and extracurricular activities. Such parental investment is partly motivated by the belief that what parents do is crucial for children’s educational success. However, this belief has not gone unchallenged. In popular books, pundits have questioned the importance of parental influence (Harris, 1998; Rowe, 1993) and lamented psychology’s focus on nurture over nature in shaping developmental outcomes (Pinker, 2002). In scientific journals, discussions continue about the relevance of parenting for children’s outcomes (Sherlock & Zietsch, 2018; Waldinger & Schulz, 2018). The debate about parental influences on children’s attainments has been fueled by three lines of evidence from behavioral genetics research. First, genetic influences have been documented for all traits and behaviors, including children’s educational attainment (Asbury & Plomin, 2014; Polderman et al., 2015). Second, children’s genetics influence the parenting they receive. This is most apparent in research reporting greater similarity in received parenting among genetically identical versus nonidentical twin children (Avinun & Knafo, 2014; Neiderhiser et al., 2004; Riemann, Kandler, & Bleidorn, 2012). Influences of children’s genetics on their received parenting come about because characteristics of children that are partly heritable elicit differences in parenting—an “evocative” gene–environment correlation (Plomin & Bergeman, 1991). Third, parents’ genetics influence the parenting they provide. This is most apparent in research documenting greater similarity in how identical versus nonidentical adult twins parent their offspring (Klahr & Burt, 2014; Neiderhiser et al., 2004). Parents’ genetics influence parenting because parenting partly reflects personal characteristics that are themselves heritable. A correlation between parents’ genetics and parenting is often referred to as a “passive” gene–environment correlation from the perspective of the child (because children will inherit genes that are correlated with the parenting to which they are exposed; Plomin, DeFries, & Loehlin, 1977). It could also be described as an “active” gene–environment correlation from the perspective of the parent, because it is a case of parents actively creating a home environment that matches their genetic dispositions. We therefore use the term “active gene–environment correlation” when referring to associations between parents’ genetics and the parenting they provide.

Gene–environment correlations in child development complicate the interpretation of socialization research (Scarr & McCartney, 1983). In particular, they raise the possibility that genetic influences confound associations between parenting and children’s educational attainment. This would be the case if genes that influence children’s educational attainment also affect the kind of parenting that is linked with educational success. Confounding could occur if parents’ education‐associated genetics shape their parenting and are also passed on to their children in whom they influence children’s educational attainment. Confounding could also occur if children’s education‐associated genetics influence both the parenting they receive and their educational attainment. In both of these scenarios, associations between parenting and children’s educational attainment may not reflect a causal effect of parenting on children. Instead, parenting may merely be a marker of parents' or children's education‐associated genetic predisposition; in theory, it is possible that parenting lacks any environmental effects on children’s educational attainment of its own (Knafo & Jaffee, 2013; Moffitt, 2005). This possibility can be summarized as “genetic confounding.”

However, gene–environment correlations do not necessarily lead to confounding. Another possibility is that the portion of parenting that is genetically influenced still affects children’s educational attainment. This would be the case if parents’ genetics influence how they parent, and parenting subsequently affects children’s educational attainment through environmental ways. Recent research provides evidence supporting this possibility, showing that education‐associated genetic variants of parents influence their children’s educational success, even if they are not passed on from parent to child (Bates et al., 2018; Kong et al., 2018). This research ruled out genetic confounding by isolating the effects of parents’ education‐associated genetic variants that were nontransmitted, that is, not passed on to children. The findings suggest that parents’ genetics influence children’s educational outcomes via environments that parents create. This possibility has been referred to as “genetic nurture” (Kong et al., 2018). It implies that treating genetics as only a confounding influence on associations between parenting and child outcomes may leave behavioral scientists with an incomplete account of parenting effects on child development.

Here we used a novel design to test gene–environment correlations, genetic confounding, and genetic nurture. Our design offers two innovative components. First, we computed genome‐wide polygenic scores for both mothers and their children using genotype data that we collected from both generations. These families are participating in the Environmental Risk (E‐Risk) Longitudinal Twin Study, a UK‐based cohort study. Polygenic scores are derived from the results of genome‐wide association studies (GWAS; Visscher et al., 2017), which scan the entire genomes of large samples of individuals to identify genetic variants associated with a phenotype. Using GWAS results as a scoring algorithm, polygenic scores aggregate the effects of genetic variants across the genome into a summary measure for an individual person. Because the focus of this study is parenting in relation to children’s educational attainment, we calculated polygenic scores based on recent GWAS of educational attainment (Lee et al., 2018). The second design innovation was that we matched molecular‐genetic data with extensive measures of mothers’ parenting that we collected in four successive family home visits during the first 12 years of children’s lives. Parenting measures were derived from multiple reporters: mothers, interview staff, and children themselves. We focused on the following aspects of parenting that have been shown to predict children’s educational attainment: cognitive stimulation; warm, sensitive parenting; low household chaos; and a safe, tidy home (Davis‐Kean, 2005; Garrett‐Peters, Mokrova, Vernon‐Feagans, Willoughby, & Pan, 2016; Spera, 2005). We measured children’s educational attainment at 18 years.

We used these data to test three hypotheses, as illustrated in Figure 1. First, we tested for the presence of gene–environment correlations. We did this by testing whether mothers’ education polygenic scores were associated with the parenting they provided (Figure 1, path a) and whether children’s polygenic scores were associated with the parenting they received (Figure 1, path b). Because biological mothers share genetics with their children (Figure 1, path c), genetic associations with parenting could either reflect active gene–environment correlations between mothers’ genetics and parenting or evocative gene–environment correlations between children’s genetics and parenting. To disentangle active from evocative gene–environment correlation, we tested whether mothers’ polygenic scores were associated with parenting after adjusting for children’s polygenic scores (indicating active gene–environment correlation) and whether children’s polygenic scores were associated with parenting after adjusting for mothers’ polygenic scores (indicating evocative gene–environment correlation). A finding of positive gene–environment correlations would indicate that education‐associated genetics shape the parenting mothers provide and children receive.

**Figure 1:**
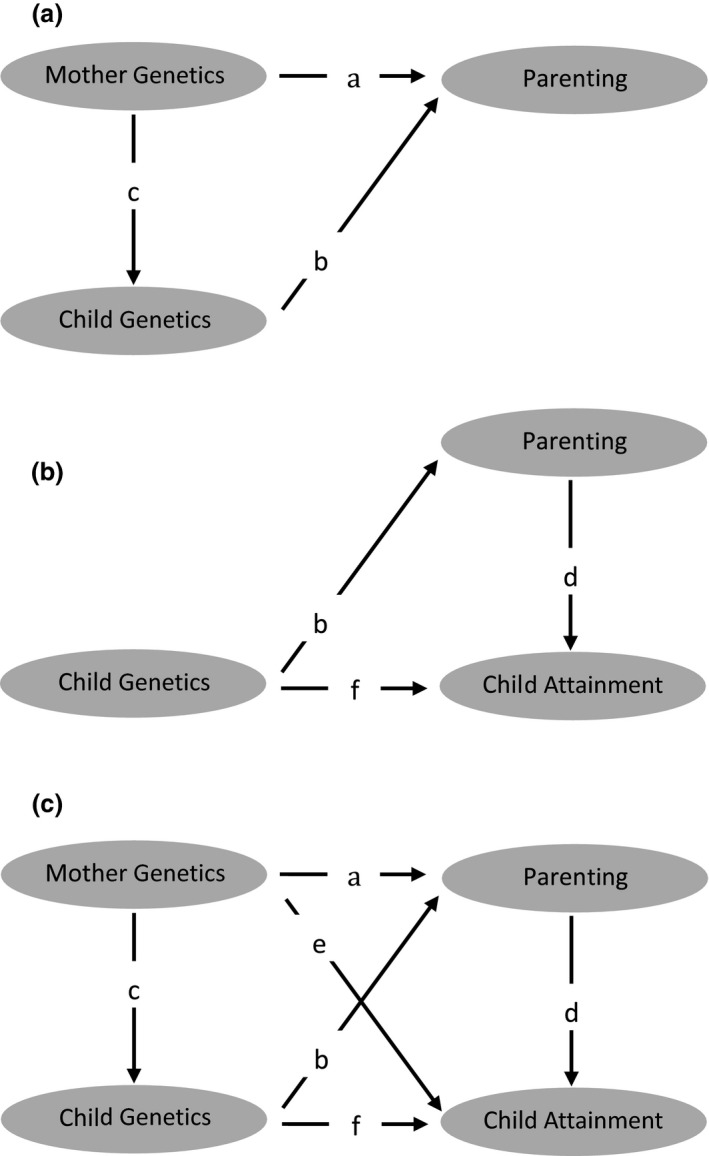
How do mothers’ and children’s education‐associated genetics influence parenting and child attainment? Testing gene environment correlation, genetic confounding, and genetic nurture. *Note.* The panels illustrate the three hypotheses tested in the present article: gene–environment correlation (panel a); genetic confounding (panel b), and genetic nurture (panel c). Panel a: Gene–environment correlations would be indicated by a nonzero path coefficient a, from mothers’ education‐associated genetics (i.e., the polygenic score) to the parenting they provide, and/or by a nonzero path coefficient b, from children’s education‐associated genetics (i.e., the polygenic score) to the parenting they receive. Panel b: Genetic confounding would be indicated by a reduction in path coefficient d between parenting and child attainment, once child genetics are controlled for. Panel c: Genetic nurture would be indicated by a nonzero partial regression coefficient of mothers' education‐associated genetics in the prediction of child attainment jointly with children’s own genetics (this analysis controls for genetic transmission of genetics that affect child attainment, i.e., paths c*f). The product of path coefficients a*d represents the part of genetic nurture mediated by measured parenting, whereas path coefficient e represents any remaining direct effect of mothers genetics’ on child attainment not mediated by measured parenting. For the purposes of this article, the path diagrams are assumed to be qualitatively correct; that is, the depicted paths are assumed to be the only ones present (although some may have zero coefficients). This assumption rules out, for example, confounding of parenting and child attainment that is not due to genetics, because such confounding is not the topic of study in this article. Note that “genetics” in this study refers to genetic influences on educational attainment as captured by the education polygenic score, which accounts for only a portion of all genetic influences on attainment. “Mother genetics” and “Child genetics” relate to the same trait, that is the polygenic score and scoring weights are the same.

Second, we tested for the presence of genetic confounding. We did this by testing whether associations between parenting and children’s educational attainment (Figure 1, path d) reduced when controlling for children’s education polygenic scores. Genetic confounding as measured using the education polygenic score is possible (a) if mothers' polygenic scores influence their parenting (Figure 1, path a) and the same genetics are passed on to children (Figure 1, path c) in whom they influence educational attainment (Figure 1, path f), or (b) if children’s polygenic scores both evoke the parenting they receive (Figure 1, path b) and also influence their educational attainment (Figure 1, path f). We therefore controlled for children’s polygenic scores to (a) control for education‐associated genetics that influence parenting in the parent generation and that are passed on to children and (b) control for education‐associated genetics in the child generation that evoke parenting (we did not additionally control for mothers’ education polygenic scores because confounding from mothers’ genetics can only arise if these genetics are passed on to children). Controlling for children’s polygenic scores does not rule out genetic confounding, because the education polygenic score measures only a portion of all genetic influences on education. However, a finding that the association between parenting and children’s education reduces after controlling for children’s education polygenic score would support the hypothesis of genetic confounding, that is, that associations between parenting and children’s educational attainment are partly influenced by the same underlying genetic disposition.

Third, we tested for the presence of genetic nurture. We did this by testing whether mothers’ polygenic scores were associated with their children’s educational attainment (Figure 1, path e). We tested this association controlling for children’s own polygenic scores, because mothers’ polygenic scores may be associated with children’s attainment simply due to biological mothers passing on genes to their children (Figure 1, path c*f). We previously reported in the E‐Risk cohort that mothers’ polygenic scores were associated with their children’s attainment over and above children’s own polygenic scores (Belsky et al., 2018). Here we directly tested the hypothesis that the parenting provided by mothers could explain this link between mothers’ polygenic scores and their children’s educational attainment (Figure 1, paths a*d). A finding that parenting explains the association would suggest that parental genetics affect children’s attainment over and above genetic transmission, via creating environments that influence children’s educational outcomes.

Our research is being done at a time when there are still many unresolved questions about polygenic scores. Two issues stand out. First, polygenic scores are noisy estimates of genetic disposition and currently account for only a small portion of all genetic influences on a phenotype. Second, GWAS do not reveal causal mechanisms linking polygenic scores to outcomes. Despite these limitations, there are several reasons why research on associations between family members’ polygenic scores and parenting is informative. First, it generates new knowledge about intergenerational transmission, by providing an opportunity to test how genetic differences observed in one generation shape the experiences and opportunities of the next generation. Second, it informs our understanding of gene–environment interplay, by challenging the notion of a dichotomy between the effects of genes and the effects of parenting on child development. Third, it sheds light on the mechanisms underpinning the associations between genetics and behavioral outcomes, by testing how genetic influences operate through environments. Previous studies indicate that genetics identified in GWAS pick up on environmental influences on the GWAS target phenotype (Kong et al., 2018; Krapohl et al., 2017; Lee et al., 2018), but there has been little systematic study of specific environmental exposures as possible mediators of genetic associations. Research about the pathways connecting genetics with behavioral outcomes is important, because the pace of GWAS discovery is accelerating. If projections materialize, polygenic scores will eventually predict a substantial portion of variability in important life outcomes; for the education polygenic score, this portion has already increased within the last 5 years from approximately 2% of variability (Rietveld et al., 2013) to approximately 10% of variability (Lee et al., 2018). Now is the time to investigate why genetics revealed in GWAS are associated with behaviors, in order to reduce the risk of misuse and misinterpretation of genetic discoveries in the future. Developmental psychologists have an opportunity to lead the way in the task of annotating GWAS findings, because they have the expertise and data to test hypotheses about developmental and social mechanisms that link genetics with outcomes (Belsky & Harden, 2019).

In summary, the goal of this article was to show how new genetic discoveries can be integrated into developmental psychology to study the questions that are of fundamental importance both to socialization and to genetics research.

## Method

### Participants

Participants were members of the E‐Risk Longitudinal Twin Study, which tracks the development of a birth cohort of 2,232 British children (Moffitt & E‐Risk Study Team, 2002). Briefly, the E‐Risk sample was constructed in 1999–2000, when 1,116 families (93% of those eligible) with same sex 5‐year‐old twins participated in home‐visit assessments. This sample comprised 56% monozygotic (MZ) and 44% dizygotic twin pairs; sex was evenly distributed within zygosity (49% male). The study sample represents the full range of socioeconomic conditions in Great Britain, as reflected in the families’ distribution on a neighborhood‐level socioeconomic index (A Classification of Residential Neighborhoods, developed by CACI, Inc., for commercial use; Odgers, Caspi, Bates, Sampson, & Moffitt, 2012; Odgers, Caspi, Russell, et al., 2012): 25.6% of E‐Risk families live in “wealthy achiever” neighborhoods, compared with 25.3% nationwide; 5.3% compared with 11.6% in “urban prosperity” neighborhoods; 29.6% compared with 26.9% in “comfortably off” neighborhoods; 13.4% compared with 13.9% in “moderate means” neighborhoods; and 26.1% compared with 20.7% in “hard‐pressed” neighborhoods. “Urban prosperity” families are underrepresented in E‐Risk because such households are often childless.

Home visits were subsequently conducted when the children were aged 7 (98% participation), 10 (96%), 12 (96%), and 18 (93%). At 18 years of age, 2,066 participants were assessed, each twin by a different interviewer. There were no differences between those who did and did not take part at age 18 in terms of socioeconomic status assessed when the cohort was initially defined (χ^2^ = .86, *p* = .65), age‐5 IQ scores (*t* = .98, *p* = .33), age‐5 behavioral or emotional problems (*t* = .40, *p* = .69 and *t* = .41, *p* = .68, respectively). The Joint South London and Maudsley and the Institute of Psychiatry Research Ethics Committee approved each phase of the study. Parents gave informed consent and twins gave assent between 5 and 12 years and then informed consent at age 18.

### Parenting

We measured the following aspects of parenting that have previously been shown to predict children’s educational attainment: cognitive stimulation; warmth and sensitivity; household chaos (reverse‐coded to indicate low household chaos); and the safety and tidiness of the family home (Table 1). These aspects of parenting were assessed during home visits conducted at four time periods (when the children were aged 5, 7, 10, and 12 years) and drew on reports averaged across multiple informants—mothers, children and interview staff—to obtain comprehensive descriptions of the parenting children experienced during the first 12 years of their lives (Table 1).

**Table 1:** Description of Parenting Measures

Measure	Age	Informant	Format	Example items/statements
Cognitive stimulation				
Activities with mother	5	Mother	12 items with “yes”/“no” responses, reliability^a^ α = .59	“Have you and the children … visited a museum?” “… been to a park?”
Child stimulation	7, 10, 12	Interviewer	Six items with “yes”/“a little”/“no” responses, mean reliability α = .75	“Do the children have toys and puzzles?” “Do the children have books?”
Warm, sensitive parenting				
Maternal warmth	5, 10	Mother	5 min speech sample^b^, interrater agreement κ = .90	“She is a delight, she is so happy, I love taking her out, she is my ray of sunshine”
Maternal dissatisfaction	5, 10	Mother	5 min speech sample^b^, interrater agreement κ=.84	“I wish I had never had her … she’s a cow, I hate her.”
Positive parenting	7, 10	Interviewer	Five items with “yes”/“a little”/“no” responses, mean reliability α = .82	“Is the parent affectionate to the child?” “Does the parent display warmth toward the child?”
Negative parenting	7, 10	Interviewer	Seven items with “yes”/“a little”/“no” responses, mean reliability α = .75	“Is the parenting of the child overly strict?” “Is the parent controlling toward the child?”
Household chaos (reverse‐coded)				
Interviewer report	7, 10, 12	Interviewer	Three items with “yes”/“a little”/“no” responses, mean reliability α = .56	“Is the house chaotic or overly noisy?” “Do the children have a predictable daily schedule?”
Mother and child report	12	Mother, child	12 items with “not”/“somewhat”/“very often or often true” responses, mean reliability α = .77	“You can hardly hear yourself think in our home” “We are always losing things at home”
Safe, tidy home	5, 7, 10, 12	Interviewer	2–4 items (depending on age) with “yes”/“a little”/“no” responses, mean reliability α = .82	“Did the home or flat appear safe?” “Are visible rooms of the house clean?”

a Internal consistency reliability (Cronbach’s alpha).

b Using procedures adapted from the 5 min speech sample method (Magaña et al., 1986) as described previously (Caspi et al., 2004).

*Cognitive stimulation* was measured when children were aged 5, 7, 10, and 12 years. At age 5, mothers responded to 12 items asking about activities with their twins (example items: “Have you and the twins visited a museum?” “… been to a park?”). Response options were “yes” or “no.” Responses were summed. The internal consistency reliability was α = .59. At ages 7, 10 and 12, study interviewers provided observations of each family’s home. After the study visit, interviewers rated homes on six items adapted from the Home Observation for Measurement of the Environment (HOME; Bradley, Caldwell, Rock, Hamrick, & Harris, 1988; Caldwell & Bradley, 1984). Items described a cognitively stimulating home environment (example items: “Do the children have toys and puzzles?” “Do the children have books?”). Response options were “yes”/“a little”/“no.” Responses were summed. Internal consistency reliabilities ranged from α = .81 to α = .70 across ages (mean reliability: α = .75).

*Warm, sensitive parenting* was measured when children were aged 5, 7, and 10 years. At ages 5 and 10, maternal warmth and dissatisfaction were each assessed using a 5 min speech sample from mothers, as previously described (Caspi et al., 2004; Magana, Goldstein, Karno, Miklowitz, & Falloon, 1986). Briefly, trained interviewers asked the mothers to speak for 5 min about each of their children (“For the next 5 min, I would like you to describe [child name] to me, what is [child name] like?”). Mothers' speech samples were audiotaped and coded by two independent trained raters (see Caspi et al., 2004 for details). Raters underwent 2 weeks of training in coding procedures, and the same rater was used to code twins in the same family. Maternal warmth (coded on a 0–5 scale) is a global measure of the whole speech sample and indexes sympathy and/or empathy toward the child (example of high warmth: “She is a delight, she is so happy, I love taking her out, she is my ray of sunshine.”). Maternal dissatisfaction (coded on a 0–5 scale) is a global measure of the whole speech sample and indexes negativism expressed about the child (example of high dissatisfaction: “I wish I had never had her … she’s a cow, I hate her.”). Interrater reliability was established by having the raters individually code audiotapes describing 40 children. Interrater agreement was *r* = .90 for maternal warmth and *r* = .84 for dissatisfaction. Raters were blind to all other E‐Risk data. At ages 7 and 10, positive and negative parenting was assessed through study interviewer observations of parent–child interactions during the study visit. After the study visit, interviewers provided ratings on items adapted from the HOME (Bradley et al., 1988; Caldwell & Bradley, 1984) and the Dyadic Parent–Child Interactive Coding System–Revised (Robinson & Eyberg, 1981; Webster‐Stratton, 1998). Five items described positive parenting (example items: “Is the parent affectionate to the child?” “Does the parent display warmth toward the child?”). Seven items described negative parenting (example items: “Is the parenting of the child overly strict?” “Is the parent controlling toward the child?”). Response options were “yes”/“a little”/“no.” Responses were summed. Internal consistency reliabilities ranged from α = .72 to α = .82 (mean reliability was α = .82 for positive parenting, and α = .75 for negative parenting).

*Household chaos* was measured when children were aged 7, 10, and 12 years. At ages 7, 10, and 12, housechold chaos was assessed through study interviewers’ observations of family’s homes. After the study visit, interviewers rated homes on three items adapted from the HOME (Bradley et al., 1988; Caldwell & Bradley, 1984; example items: “Is the house chaotic or overly noisy?”; “Do the children have a predictable daily schedule?”). Response options were “yes”/“a little”/“no.” Responses were summed. Internal consistency reliabilities ranged from α = .53 to α = .58 across ages (mean reliability α = .56). At age 12, housechold chaos was assessed through reports from mothers and children. Mothers and children responded to the same 12 items, which were adapted from the Confusion, Hubbub, and Order Scale (CHAOS; (Matheny, Wachs, Ludwig, & Phillips, 1995), the Family Routines Inventory (Jensen, James, Boyce, & Hartnett, 1983) and the Family Ritual Questionnaire (Fiese & Kline, 1993) following previous research (Evans, Gonnella, Marcynyszyn, Gentile, & Salpekar, 2005; example items: “You can hardly hear yourself think in our home”; “We are always losing things at home”). Response options were “not”/“somewhat”/“very often or often true.” Responses were summed. Internal consistency reliabilities were α = .76 for mother’s report and α = .78 for children’s report.

*Safety and tidiness of the home* were measured when children were aged 5, 7, 10 and 12 years, through study interviewer observations of family’s homes. After the study visit, interviewers rated homes on two to four items (depending on age) adapted from the HOME (Bradley et al., 1988; Caldwell & Bradley, 1984; example items: “Did the home or flat appear safe?”; “Are visible rooms of the house clean?”). Response options were “yes”/“a little”/“no.” Responses were summed. Internal consistency reliabilities ranged from α = .77 to α = .88 across ages (mean reliability: α = .82).

To create a summary measure for each aspect of parenting, we adopted the following approach: first, we standardized each measure to *M* = 0, *SD* = 1. Second, we reverse‐coded measures so that they were in the same direction within each aspect of parenting (e.g., within warm, sensitive parenting, the measure of maternal dissatisfaction was reverse‐coded to indicate low dissatisfaction). Third, we averaged scales across different informants within age. Fourth, we averaged measures across ages. We standardized each measure to *M* = 0, *SD* = 1.

Parenting summary measures were positively correlated with each other (Table S1; mean correlation *r* = .60, range .44–.72, all statistically significant at *p *< .01).

### Children’s Educational Attainment

Children’s educational attainment was assessed in the age‐18 interview, when children were asked to report their highest educational achievement. Educational attainment was classed following the Qualification and Credit Framework, a credit‐based system used in the United Kingdom to assign educational qualifications to a set of ranked levels (http://www.accreditedqualifications.org.uk/qualifications-and-credit-framework-qcf.html). Eighteen‐year‐olds were classed: as Level 0 if they had no educational qualifications (3.4%); as Level 1 if they scored a grade of D–G on their General Certificate of Secondary Education (GCSE; 18.5%); as Level 2 if they scored a grade of A*–C (29.3%); and as Level 3 if they had achieved or were currently working towards university entrance level qualifications (or equivalent; 48.9%).

### Genotyping and Imputation

We used Illumina HumanOmni Express 24 BeadChip arrays (Versions 1.1 and 1.2; Illumina, Hayward, CA) to assay common single‐nucleotide polymorphism (SNP) variation in the genomes of E‐Risk participants and their mothers. We imputed additional SNPs using the IMPUTE2 software (Version 2.3.1, https://mathgen.stats.ox.ac.uk/impute/impute_v2.html; Howie, Donnelly, & Marchini, 2009) and the 1,000 Genomes Phase 3 reference panel (Abecasis et al., 2012). Imputation was conducted on SNPs appearing in dbSNP (Version 140; http://www.ncbi.nlm.nih.gov/SNP/; Sherry et al., 2001) that were “called” in more than 98% of the samples. Invariant SNPs were excluded. The E‐Risk cohort contains MZ twins, who are genetically identical; we therefore empirically measured genotypes of one randomly selected twin per pair and assigned these data to their MZ co‐twin. Prephasing and imputation were conducted using a 50‐million‐base‐pair sliding window. The resulting genotype databases included genotyped SNPs and SNPs imputed with 90% probability of a specific genotype among European‐descent members of the E‐Risk cohort. We analyzed SNPs in Hardy–Weinberg equilibrium (*p *> .01). We restricted our analyses to European‐descent study participants because allele frequencies, linkage disequilibrium patterns, and environmental moderators of associations may vary across populations (Martin et al., 2017). Of the *N* = 1,116 E‐Risk families, there were *n* = 860 families for whom family members’ genetic data could be analyzed, based on the mothers and at least one child having genetic data. In families with and without genetic data, there were no differences in parenting, but children’s educational attainment tended to be lower among those for whom genetic data were analyzed (*p* = .05).

### Polygenic Scoring

The polygenic scoring method uses GWAS results as a scoring algorithm to aggregate the effects of genetic variants across the genome into a single score for an individual person. We conducted polygenic scoring following the method described by Dudbridge (2013), using the polygenic scoring software PRSice (Euesden, Lewis, & O’Reilly, 2015). Briefly, we used publicly available summary statistics from the most recent GWAS of educational attainment (Lee et al., 2018), released by the Social Science Genetic Association Consortium (https://www.thessgac.org/data). This GWAS is based on analyses of approximately 1.1 million individuals of European‐descent, from 71 cohorts. The cohorts cover different countries (e.g., UK, USA, Iceland) and different historical periods (the range in birth year is approximately 1901–1989, Lee et al., 2018). Summary statistics from the GWAS report the direction and magnitude of the association between each SNP tested in the GWAS and educational attainment. This information was matched with SNPs in our E‐Risk database. For each SNP, the count of education‐associated alleles was weighted according to the effect estimated in the GWAS. Weighted counts were averaged across SNPs to compute polygenic scores. There are two issues to consider when computing polygenic scores. First, polygenic scoring is sometimes restricted to a subset of SNPs; for example, SNPs that reach genome‐wide significance in the GWAS, or SNPs that are not in linkage disequilibrium with one another (i.e., statistically independent SNPs). Theory, simulation, and empirical evidence suggest that polygenic scores are best constructed using data from all available SNPs (Dudbridge, 2013; Ware et al., 2017; Wray, Goddard, & Visscher, 2007). We therefore used all the matched SNPs to compute polygenic scores, irrespective of nominal significance for their association with educational attainment and linkage disequilibrium between SNPs. Second, polygenic scores are sensitive to bias arising from differences in allele‐frequency between populations of different ancestry (Martin et al., 2017). This is referred to as population stratification. Polygenic‐score analysis is therefore typically conducted within populations of the same genetic ancestry (e.g., individuals of European ancestry), and the analysis usually includes covariate adjustment for principal components estimated from genome‐wide SNP data to account for any residual population stratification (Price et al., 2006). The GWAS on which our polygenic score is based was conducted in individuals of European descent, and we restricted our analyses to study participants of European descent. We also conducted a principal components analysis of our genome‐wide SNP database using PLINK v1.9 (Chang et al., 2015) to control for possible residual population stratification. We residualized polygenic scores for the first ten principal components estimated from the genome‐wide SNP data. The residualized score was normally distributed and standardized to *M* = 0, *SD* = 1.

### Statistical Analysis

We used structural equation models for dyads with indistinguishable members (Kenny, Kashy, & Cook, 2006) to test gene–environment correlation, genetic confounding, and genetic nurture. In these models, analyses are conducted at the family level while constraining means and corresponding paths for twins to be equal. To test gene–environment correlation, we fitted a model predicting parenting from mothers’ and children’s education polygenic scores, first each separately, then all together in the same model. To test genetic confounding, we fitted a model predicting children’s educational attainment from parenting, and tested whether associations between parenting and educational attainment reduced when accounting for children’s polygenic score. To test genetic nurture, we fitted a model predicting children’s educational attainment from mothers’ education polygenic score, and added children’s polygenic score to this model to test the effects of mothers’ polygenic score over and above children’s own score. We then added the parenting variables to this genetic nurture model as mediators. Each parenting variable was initially tested separately, and then all mediators were entered together into the same model. Path estimates for all models as well as measures of variance explained in the outcome variables (*R* ^2^) are provided in Table S2. We adjusted for children’s sex in all analyses. All analyses were conducted using Mplus version 8.2 (Muthén & Muthén, 1998–2017).

## Results

### Both Nature and Nurture Predict Children’s Educational Attainment

**Figure 2:**
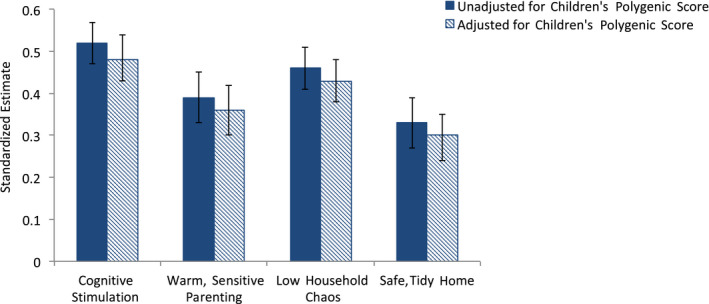
Genetic confounding: Controlling for children’s polygenic scores slightly reduces associations between parenting and children’s educational attainment. *Note.* The bars indicate the estimates (expressed as standardized regression coefficients) of predicting children’s educational attainment from parenting. Error bars indicate 95% confidence intervals. All analyses are adjusted for children’s sex. [Color figure can be viewed at wileyonlinelibrary.com]

As expected, children’s education polygenic scores were associated with their educational attainment: children with higher polygenic scores completed higher levels of education (β = .26 [95% CI .22, .31], *p* < .01). Also as expected, parenting was associated with children’s educational attainment: children exposed to greater cognitive stimulation, more warm, sensitive parenting, less household chaos, and a safer, tidier home environment went on to complete more education (estimates ranged from β = .33 for safe, tidy home environment to β = .52 for cognitive stimulation; Figure 2).

### Testing Gene–Environment Correlation: Are Mothers’ and Children’s Education Polygenic Scores Associated With Parenting?

**Figure 3:**
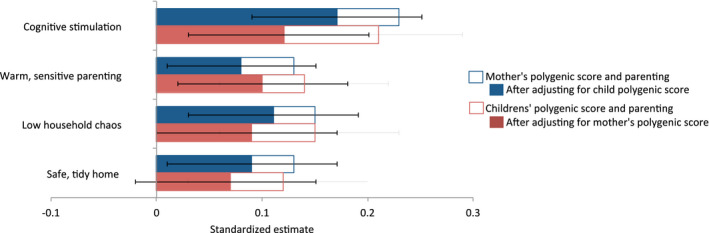
Gene–environment correlation: Mothers’ and children’s education polygenic scores are associated with parenting. *Note.* The bars indicate the estimates (expressed as standardized regression coefficients) of predicting parenting from mothers’ and children’s education polygenic scores. Error bars indicate 95% confidence intervals. Analyses are adjusted for children’s sex. [Color figure can be viewed at wileyonlinelibrary.com]

Our results provided evidence for gene–environment correlation. Mothers with higher education polygenic scores provided greater cognitive stimulation and more warm, sensitive parenting, and raised their children in less chaotic and safer, tidier homes (estimates ranged from β = .13 for safe, tidy home environment to β = .23 for cognitive stimulation; Figure 3). Children with higher polygenic scores also received more cognitive stimulation and more warm, sensitive parenting, and were raised in less chaotic and safer, tidier homes (estimates ranged from β = .12 for safe, tidy home to β = .21 for cognitive stimulation; Figure 3). As would be expected based on biological mothers passing on genes to their children, mothers’ and children’s education polygenic scores were correlated (β = .52 [95% CI .47, .57], *p* < .01), We therefore included mothers’ and children’s education polygenic scores in the same models when predicting parenting. In these models, mothers’ education polygenic scores were associated with all aspects of parenting independently of their children’s polygenic scores, indicating active gene–environment correlations between mothers’ genetics and parenting (Figure 3). In addition, children’s polygenic scores were associated with cognitive stimulation, warm, sensitive parenting, and low household chaos independently of their mothers’ polygenic scores, indicating evocative gene–environment correlations between children’s genetics and these aspects of parenting (Figure 3).

### Testing Genetic Confounding: Do Genetic Influences Confound Associations Between Parenting and Children’s Education?

Our results provided modest evidence for genetic confounding. Without controlling for genetics, children exposed to greater cognitive stimulation, more warm, sensitive parenting, less household chaos, and a safer, tidier home environment went on to complete more education (estimates ranged from β = .33 for safe, tidy home environment to β = .52 for cognitive stimulation; Figure 2). Controlling for children's polygenic scores led to a significant reduction of these associations, by approximately 8% (Figure 2), but the attenuations were small and parenting continued to be a statistically significant predictor of educational attainment. These findings indicate that genetic influences, as captured by the education polygenic score, account for only a small part of the reason for why parenting is associated with children’s educational attainment.

### Testing Genetic Nurture: How Do Maternal Genetics and Parenting Combine to Influence Children’s Education?

Our findings provided evidence for genetic nurture. We first tested whether mothers’ education polygenic scores were associated with their children’s educational attainment; this was the case (β = .22 [95% CI .16, .28], *p* < .01). The association was not entirely due to mothers passing on education‐associated genetics to their children; mothers’ education polygenic scores predicted their children’s educational attainment over and above children’s own polygenic scores (adjusted β = .11 [95% CI .04, .18], *p* < .01). This finding suggests the hypothesis that mothers’ education‐associated genetics shape family environments that affect children’s attainments independently of mother–child genetic transmission. We tested this hypothesis by adding measures of parenting to the analysis model. Of the four parenting measures, three (cognitive stimulation, household chaos, and a safe, tidy home) emerged as statistically significant (*p* < .05) mediators (Table 2). Cognitive stimulation on its own accounted for 71% of the association between maternal education‐associated genetics and children’s educational attainment. Low household chaos and a safe, tidy home each mediated 39% and 22% of the association, respectively, but in a model containing cognitive stimulation, only low household chaos accounted for a small portion of additional covariance beyond cognitive stimulation. These findings indicate that mothers’ education polygenic scores are associated with their children’s attainment via mothers’ parenting, particularly the extent of cognitive stimulation mothers provided to their children.

**Table 2:** Genetic Nurture: Parenting Mediates Associations Between Mothers’ Education Polygenic Scores and Their Children’s Educational Attainment Independently of Children’s Polygenic Scores

	Parenting				
	Cognitive stimulation	Warm, sensitive parenting	Low household chaos	Safe, tidy home	All mediators together
	Estimate (95% CI)	Estimate (95% CI)	Estimate (95% CI)	Estimate (95% CI)	Estimate (95% CI)
Total effect	**.11 (.04, .18)**	**.11 (.04, .18)**	**.11 (.04, .18)**	**.11 (.04, .18)**	**.11 (.04, .18)**
Direct effect	.03 (−.03, .09)	**.09 (.02, .15)**	**.07 (.00, .13)**	**.09 (.02, .15)**	.03 (−.03, .09)
Indirect effect	**.08 (.04, .12)**	.02 (−.01, .05)	**.04 (.01, .08)**	**.02 (.00, .05)**	**.08 (.04, .12)**
% Mediation	**71**	20	**39**	**22**	**70**

Note

The table shows results of analyses testing whether the different aspects of parenting we measured (cognitive stimulation; warm, sensitive parenting; low household chaos; safe, tidy home) mediated associations between mothers’ education polygenic scores and their children’s educational attainment independently of children’s polygenic scores. Each column reports results from a model testing a different mediator; the last column reports results from a model testing all mediators jointly. Within each column, the “total effect” is an estimate of the association before adding the parenting mediator(s); this does not differ across models. The “direct effect” is an estimate of how much of the association remains after adding the parenting mediator(s; corresponding to path e in Figure 1). The “indirect effect” is an estimate of the amount of mediation through the parenting mediator(s); expressed as a percentage in the row “% Mediation” (corresponding to paths a*d in Figure 1). All estimates are standardized. Bolded estimates indicate statistically significant (*p* < .05) effects. 95% confidence intervals (CI) were obtained from 1,000 bootstrap replications.

## Discussion

The investments parents make to raise their offspring are thought to be a major contributor to children’s educational success, making parental investment a cornerstone of psychological, sociological, and economic models that seek to explain how educational inequalities are created and perpetuated (Cheng, Johnson, & Goodman, 2016; Feinstein, Duckworth, & Sabates, 2004; Kalil, 2015). However, findings from behavioral‐genetic studies have challenged causal interpretations of parental influence by showing genetic influences on parenting; a gene–environment correlation. Here we tested implications of gene–environment correlations for parental investment in children’s educational attainment using a novel design—in a prospective‐longitudinal study, we collected genotype data from both mothers and children and matched these genetic data with home‐visit measures of parenting behavior. We report three main findings.

First, we found evidence for gene–environment correlations. Both mothers’ and children’s education‐associated genetics, summarized in genome‐wide polygenic scores, were associated with the kind of parenting that is known to be linked with children’s later educational success. By collecting genetic data from both mothers and their offspring, we were able to show that different forms of gene–environment correlations operate in the same family, at the same time. Both active and evocative gene–environment correlations were implicated in the cognitive stimulation and the warm, sensitive parenting that children experienced and in the kinds of households (chaotic; safe and tidy) in which children grew up. Second, we found evidence for slight genetic confounding. The estimated effects of mothers’ parenting on children’s educational attainment were significantly reduced after accounting for education‐associated genetics, consistent with a view of genes as confounding part of the link between parenting and child attainment. However, the magnitude of confounding as measured using the polygenic score was small. Third, we found evidence for genetic nurture. Parenting behavior—particularly mothers’ cognitive stimulation of their children—explained why mothers’ genetics were associated with their children’s educational attainment (independently of children's own genetics). This finding extends recent reports of associations between parental genetics and children’s educational attainment (Bates et al., 2018; Belsky et al., 2018; Kong et al., 2018; Liu, 2018) by showing, for the first time, that parents’ education‐associated genetics shape the features of the family environment that predict the next generation’s educational success.

Our findings need to be interpreted in light of several limitations. First, our approach to estimating genetic nurture relies on the assumption that mothers’ and children’s polygenic scores are measured with identical error. To the extent that this assumption is violated, our estimates of genetic nurture could be upwardly or downwardly biased, depending on whether error is greater in mothers’ versus children’s polygenic scores. However, the assumption is probably defensible, because mothers’ and children’s polygenic scores are identical measurements, that is, sums of the same genotypes transformed using the same weights. Second, although the education polygenic score that we used is based on the largest‐ever social science GWAS, a limitation of this GWAS is that it still reflects only a portion of all genetic influences on educational attainment (approximately one third; Lee et al., 2018). To the extent that the polygenic score is an underestimate of the total genetic influence on educational attainment, our estimates of gene–environment correlations, genetic confounding, and possibly genetic nurture are likely to be underestimates of the true effects. At this point, our findings provide “proof‐of‐principle” of these processes, and the implications they raise can continue to be tested as refined polygenic scores become available. Third, we tested genetic confounding and genetic nurture only for children’s educational attainment, not for other child outcomes. We focused on educational attainment because it is a central determinant of future health, wealth, and well‐being (Cutler & Lleras‐Muney, 2010; Hout, 2012; Oreopoulos & Salvanes, 2011), and because the polygenic score for educational attainment is based on the largest GWAS of a social‐behavior phenotype (Lee et al., 2018). As increasingly larger GWAS are conducted for more developmental outcomes, the same design we present here can be used to test genetic confounding and genetic nurture for other outcomes. Fourth, our study members are still young and most of them have not yet completed their final educational degree. Our measure of educational attainment is therefore only a proxy measure. However, UK students’ qualifications obtained by age 18 are good indicators of their educational pathways beyond age 18 (UK Department for Education, 2018). Furthermore, polygenic‐score associations with educational attainment are very similar between our study and studies of adults (Belsky et al., 2018; Lee et al., 2018), and findings of genetic nurture have been observed in studies of adults who have completed their education (Bates et al., 2018; Belsky et al., 2018; Kong et al., 2018; Lee et al., 2018). Fifth, our labeling of correlations between parents’ genetics and parenting as “active gene–environment correlation” may elicit skepticism among some readers, because correlations between genetics and parenting are typically examined from the perspective of the child and are then referred to as “passive” gene–environment correlations. We would argue that a correlation between parents’ genes and parenting qualifies as active gene–environment correlation, which has been defined as a person contributing to their own environment, and actively seeking an environment related to their genetic propensities (Plomin et al., 1977). Specifically, the aspects of parenting we examine—the cognitively stimulating activities that parents and children engage in together; the warmth and sensitivity of the parent–child relationship; the chaos in the household; and the safety and tidiness of the home—all represent environmental exposures for the parent and the child, that are actively created and shaped by parents to match their genetic dispositions. This interpretation of active gene–environment correlation also follows the concept of niche construction in animal ecology, whereby organisms actively modify their own and each others’ environments; for example by building nests for their offspring (Odling‐Smee, Laland, & Feldman, 2003). Sixth, there is a wide variety of measures available to study parenting and our findings may not generalize to all of these other measures. However, we recently reported very similar associations to the ones observed in our study in an independent sample, using measures of parenting that were derived using what some researchers view as the “gold standard” of parenting assessment—observer ratings of videotaped parent–child interactions (Wertz et al., 2019). The replication across two independent cohorts and different measurements of parenting bolsters the substance of our findings. Seventh, we did not have genetic data from fathers, which means that we were unable to control for fathers’ education polygenic scores when estimating associations between mothers’ and children’s education polygenic scores and parenting. To the extent that fathers’ genes are correlated with parenting, the associations we observed in our study may partly reflect effects of fathers’ genetics, because biological fathers’ and children’s genes are correlated (due to genetic inheritance) and because mothers’ and fathers’ genetics may be correlated (due to assortative mating, i.e., the tendency to select partners with characteristics similar to one’s own). Eigth, even though our research is genetically informative, it is still observational, and hence cannot establish causal relationships between genetics, parenting, and children’s educational attainment. What it can do is (a) point to pathways through which genetic influences may contribute to intergenerational transmission; (b) elucidate processes of gene–environment interplay in parenting and child development; (c) shed light on possible developmental and social mechanisms that link parent and child education‐associated genetics with future attainment; and (d) provide an example of how to integrate new genomic discoveries into developmental psychology to study questions relevant to child development. Against this background, we conclude by discussing the implications of our findings about gene–environment correlations, genetic confounding, and genetic nurture for a more thorough understanding of the developmental processes that shape children’s attainment.

Our findings of gene–environment correlation replicate and extend our prior work on genetic associations with parenting (Wertz et al., 2019). We replicated findings from a previous analysis in a New Zealand cohort, in which we showed that parents’ education polygenic scores were associated with the warm, sensitive, stimulating parenting they provided to their children (Wertz et al., 2019). Here we report the same pattern of results in an independent cohort of British mothers, indicating that genetic correlations with parenting are robust against differences in context and measurements of parenting. We extend this prior work by incorporating children’s polygenic scores in our analyses, finding that children’s genetics are associated with the parenting they receive. Together with other recent studies (Dobewall et al., 2018; Krapohl et al., 2017; Selzam et al., 2018), these findings provide molecular‐genetic evidence for a bidirectional model of parent–child relations, in which parenting is partly a response to children’s characteristics (Bell, 1968; Crouter & Booth, 2003; Pardini, 2008; Sameroff, 2010).

Findings of gene–environment correlations with parenting imply that the family environments children experience while growing up are partly a function of their own and their parents’ genetics. For example, we found that children of parents who carried a higher number of education‐associated variants were exposed to greater cognitive stimulation in the home compared to children of parents who carried fewer of these variants. Because biological parents and children share genes, family environments shaped by parents’ genes will tend to match and reinforce children’s genetic dispositions (Knafo & Jaffee, 2013; Scarr & McCartney, 1983; Tucker‐Drob & Harden, 2012). Such a match can positively influence children’s development; for example, when a child with a high education polygenic score is born into a family that provides cognitive stimulation. However, the same match also implies that a children with lower education polygenic scores will tend not to experience exactly the kind of stimulating and supportive parenting that could make a difference for their attainment. Thus, for better and for worse, correlations between genes and environments can reduce the availability of experiences that alter individuals’ developmental trajectories. This also applies to the reproduction of educational success across generations. To the extent that educational outcomes are influenced by genetics, genes will tend to be a force for intergenerational stability in educational attainment, both via direct genetic transmission and via indirect effects of genes on caregiving environments that shape future generations’ behaviors. This tendency means that is it important to improve children’s access to interventions that may be able to break reinforcing links between genes and environments, such as high‐quality early skill‐building programs (Heckman, 2006).

Given how much attention critics of parenting effects devote to the possibility of genetic confounding (Harris, 1998; Rowe, 1993; Sherlock & Zietsch, 2018), it may seem surprising that our estimates of genetic confounding were so small. There are two possible explanations for this finding: either genetics do little to confound associations between parenting and children’s educational attainment, or we have underestimated the true magnitude of genetic confounding. The observation that polygenic‐score associations with educational attainment are substantially lower than heritability estimates of educational attainment (Branigan, McCallum, & Freese, 2013) suggests that our findings underestimate genetic confounding. Currently, even the best and biggest efforts to capture the genetic variants associated with educational attainment are still missing a substantial part of its heritability (Manolio et al., 2009; National Human Genome Research Institute, 2018). Until more of this “missing heritability” can be accounted for at the molecular‐genetic level, the safest way to rule out genetic confounding is to continue to use family‐based designs, such as discordant‐twin designs (McGue, Osler, & Christensen, 2010; Vitaro, Brendgen, & Arseneault, 2009), parent–child adoption designs (Leve et al., 2013) or children‐of‐twin designs (D’Onofrio et al., 2003), that can estimate associations between parenting and children’s educational attainment free from genetic influences shared between parents and children (Turkheimer & Harden, 2014).

Debates about parental influences on children’s development tend to contrast the effects of parents’ genes—assumed to influence children via genetic transmission—with the effects of parenting—assumed to influence children via environmental ways. Our finding of genetic nurture draws a more nuanced picture, by showing that mothers’ genetics were associated with children’s attainment over and above genetic transmission, via parenting. This finding has three implications. First, over and above a persons’ own genetics, their development will be shaped by the genetics of significant others. We demonstrate this here for effects of parents’ genetics on children’s outcomes, but this observation likely extends beyond parents to everyone who creates environments inhabited by people: family members; individuals residing outside the family context, such as peers and partners (Conley et al., 2016; Domingue et al., 2018); even people to whom a child may be exposed to only indirectly, such as the grandparents who raised a child’s parents (Hällsten & Pfeffer, 2017; Kong et al., 2018; Liu, 2018). The existence of a “social genome” broadens the scope of the study of genetics, from an individual’s genes and their effects on an individual’s phenotype, to the genomes of the individuals making up an individual’s social context (Domingue & Belsky, 2017). Second, much has been written about the need to integrate genetics into parenting research and socialization theory, but there is also a need to integrate environments into how we think about and collect genetic data. Correlations between genes and environments are a challenge not only for socialization research, but also for genetics research: Although DNA sequence cannot be modified by the environment, our findings show that environments still pose a threat to causal inference, because associations between a person’s DNA and developmental outcomes may partly reflect effects of environments created through genes of other individuals (Bates et al., 2018; Kong et al., 2018). As much as genetic confounding needs to be considered when estimating environmental effects, “environmental confounding” needs to be taken into account when estimating genetic effects (Krapohl et al., 2017; Young et al., 2018). Third, environments are part of the pathway from genotype to phenotype (Kandler & Zapko‐Willmes, 2017; Scarr & McCartney, 1983). Specifically, we found that genetic influences on children’s educational attainment partly manifested through parenting; an environmentally mediated genetic effect. The finding shows that new GWAS discoveries are not inimical to socialization theories, because these genetics partly work through factors that socialization researchers have studied for decades, such as the home environment. Combining genetic data with measures of individuals’ social environments is key to tracing how genetics affect life outcomes. By joining forces in this way, genetics and socialization researchers will be able to strengthen causal estimates and obtain a more complete understanding of the processes shaping children’s attainments.

## Supporting information


**Table S1.** Correlations Between Summary Measures of Parenting

**Table S2.** Standardized Path Estimates for the Paths Contained in the “Gene–Environment Correlation,” “Genetic Confounding,” and “Genetic Nurture” Models (as Depicted in Figure 1; Main Manuscript), As Well As *R* ^2^ Values for Dependent Variables in Each Model
