# Supplementary material for: Using DNA From Mothers and Children to Study Parental Investment in Children’s Educational Attainment
Source: Child Dev. 2019 Oct 27;91(5):1745–61. doi: 10.1111/cdev.13329 (PMC7183873; doi:10.1111/cdev.13329)
Supplement: Supplementary file 1 — Table S1. Correlations Between Summary Measures of Parenting [file CDEV-91-1745-s001.docx]

**Supplementary Table 1.** The table reports correlations between summary measures of parenting.

|  | Cognitive stimulation | Warm, sensitive parenting | Low household chaos |
| --- | --- | --- | --- |
| Cognitive stimulation | 1 |  |  |
| Warm, sensitive parenting | .58 | 1 |  |
| Low household chaos | .72 | .60 | 1 |
| Safe, tidy home | .63 | .44 | .63 |
