# Supplementary material for: Using DNA From Mothers and Children to Study Parental Investment in Children’s Educational Attainment
Source: Child Dev. 2019 Oct 27;91(5):1745–61. doi: 10.1111/cdev.13329 (PMC7183873; doi:10.1111/cdev.13329)
Supplement: Supplementary file 2 — Table S2. Standardized Path Estimates for the Paths Contained in the “Gene–Environment Correlation,” “Genetic Confounding,” and “Genetic Nurture” Models (as Depicted in Figure 1; Main Manuscript), As Well As R 2 Values for Dependent Variables in Each Model [file CDEV-91-1745-s002.docx]

**Supplementary Table 2**. The table reports standardized path estimates for the paths contained in the ‘gene-environment correlation’; ‘genetic confounding’; and ‘genetic nurture’ models (as depicted in Figure 1; main manuscript), as well as R^2^ values for dependent variables in each model.

|  | Parenting | | | |
| --- | --- | --- | --- | --- |
|  | Cognitive stimulation | Warm, sensitive parenting | Low household chaos | Safe, tidy home |
| **Gene-environment correlation** | β (95%CI) | β (95%CI) | β (95%CI) | β (95%CI) |
| Path a (mom pgs -> parenting) | .17 (.09, .25) | .08 (.01, .15) | .11 (.03, .19) | .09 (.01, .17) |
| Path b (child pgs -> parenting) | .12 (.03, .20) | .10 (.02, .18) | .09 (.00, .17) | .07 (-.02, .15) |
| Path c (mom pgs -> child pgs) | .52 (.47, .57) | .52 (.47, .57) | .52 (.47, .57) | .52 (.47, .57) |
| R^2^ (in child polygenic score) | 27.0% | 27.0% | 27.0% | 27.0% |
| R^2^ (in parenting) | 7.0% | 3.5% | 3.9% | 2.1% |
| **Genetic confounding** |  |  |  |  |
| Path b (child pgs -> parenting) | .22 (.15, .29) | .14 (.07, .20) | .15 (.08, .22) | .14 (.06, .21) |
| Path f (child pgs -> attainment) | .18 (.12, .23) | .23 (.18, .29) | .22 (.16, .27) | .24 (.19, .30) |
| Path d (parenting -> attainment) | .48 (.43, .54) | .36 (.30, .42) | .43 (.38, .48) | .30 (.24, .35) |
| R^2^ (in attainment) | 30.8% | 19.7% | 26.1% | 17.3% |
| R^2^ (in parenting) | 5.2% | 3.2% | 3.5% | 1.9% |
| **Genetic nurture** |  |  |  |  |
| Path a (mom pgs -> parenting) | .16 (.08, .24) | .06 (-.02, .14) | .10 (.02, .19) | .08 (.00, .16) |
| Path b (child pgs -> parenting) | .13 (.04, .21) | .10 (.03, .18) | .09 (.02, .17) | .08 (.00, .18) |
| Path c (mom pgs -> child pgs) | .52 (.46, .58) | .52 (.46, .58) | .52 (.46, .58) | .52 (.46, .58) |
| Path d (parenting -> attainment) | .48 (.43, .52) | .35 (.29, .42) | .42 (.37, .48) | .29 (.23, .35) |
| Path e (mom pgs -> attainment) | .03 (-.03, .09) | .09 (.02, .15) | .07 (.00, .13) | .09 (.02, .15) |
| Path f (child pgs -> attainment) | .16 (.10, .22) | .19 (.12, .25) | .18 (.12, .25) | .20 (.13, .27) |
| R^2^ (in child polygenic score) | 27.0% | 27.0% | 27.0% | 27.0% |
| R^2^ (in attainment) | 30.6% | 19.8% | 26.2% | 17.5% |
| R^2^ (in parenting) | 7.0% | 3.3% | 4.1% | 2.3% |

*Note:* All models include sex as a covariate. β= Standardized path estimate; CI=Confidence interval. The ‘gene-environment correlation’ model includes families with valid data for mother and children polygenic scores and for parenting (n=857). The ‘genetic confounding’ and ‘genetic nurture’ models include families that additionally have valid data for child attainment (n=821).
